# A reference dataset for verifying numerical electrophysiological heart models

**DOI:** 10.1186/1475-925X-10-11

**Published:** 2011-01-27

**Authors:** Hans Koch, Ralf-Dieter Bousseljot, Olaf Kosch, Cosima Jahnke, Ingo Paetsch, Eckart Fleck, Bernhard Schnackenburg

**Affiliations:** 1 Physikalisch-Technische Bundesanstalt (PTB), Abbestr. 2-12, 10587 Berlin, Germany; 2 Deutsches Herzzentrum Berlin (DHZB, German Heart Institute Berlin), Augustenburger Platz 1, 13353 Berlin, Germany; 3 Philips GmbH Healthcare, Lübeckertordamm 5, 20099 Hamburg, Germany

## Abstract

**Background:**

The evaluation, verification and comparison of different numerical heart models are difficult without a commonly available database that could be utilized as a reference. Our aim was to compile an exemplary dataset.

**Methods:**

The following methods were employed: Magnetic Resonance Imaging (MRI) of heart and torso, Body Surface Potential Maps (BSPM) and MagnetoCardioGraphy (MCG) maps. The latter were recorded simultaneously from the same individuals a few hours after the MRI sessions.

**Results:**

A training dataset is made publicly available; datasets for blind testing will remain undisclosed.

**Conclusions:**

While the MRI data may provide a common input that can be applied to different numerical heart models, the verification and comparison of different models can be performed by comparing the measured biosignals with forward calculated signals from the models.

## Background

Cardiac modeling has been successfully applied in basic research to advance our understanding of fundamental electrophysiological mechanisms in health and disease. While this discipline is rapidly growing, it is becoming more and more difficult to evaluate the quality of the models generated. In order to be able to compare different models, it is a prerequisite to apply them at least to the same heart morphologies.

Therefore, it was a major step forward when the so-called "San Diego rabbit heart" and "San Diego canine heart" of the Cardiac Mechanics Research Group of the UCSD [1] achieved the status of a kind of reference. Due to the open access policy and its high resolution geometrical data, it became a popular workhorse for the modeling community. Particularly the data of fiber orientations were of significant value and boosted the quality of modeling, because the anisotropy could be considered in a realistic manner.

Other very ambitious approaches, e.g. by the Center for Cardiovascular Bioinformatics and Modeling at JHU [2] or by collaboration between groups in Oxford, UK, and Graz, Austria [3], further improve the database to aid more realistic heart modeling.

However, the final goal should be the human equivalent to the above-mentioned reference hearts, and the first of those start to appear (c.f. [2]). A common practice has been the use of publicly available data of the visible human project^® ^of the US National Library of Medicine [4]. The drawback is the comparatively low spatial resolution which means that fiber orientation data cannot be extracted easily. On the other hand, the torso geometries are available and could be used favorably to include the volume conductor environment to heart modeling.

A major disadvantage of the above-mentioned heart datasets is that they are ex-vivo, i.e. they lack the information of a realistic dynamic geometry change. Simple model calculation demonstrated greatly differing outcomes for the electrophysiological activation propagation and the resulting ElectroCardioGram (ECG) and/or MagnetoCardioGram (MCG), depending on whether the systole or the diastole geometry was implemented in the algorithm [5]. In addition, ex-vivo geometries may differ considerably from the in-vivo state.

In recent years, the inclusion of electromechanical coupling in heart modeling has become a popular challenge [6,7]. Thus standard human heart geometry with realistic dynamics would be of great value. With progress in imaging technology using the so-called CINE or tagged Magnetic Resonance Imaging (MRI) [8,9], such data can be provided. However, until now no dynamic human heart dataset has been established. But even if such a reference dataset did exist, it would only provide a common comparable input to different models. In addition, the reference dataset has to be tested for its common applicability. One realistic criterion could be the measured biosignals of the same person from whom the MRI images are taken.

If the heart models are extended in such a way as to calculate the ECG in a forward manner from the model, then a comparison between measured and calculated ECGs could provide a measure for the discrepancy between model and reality.

To improve representativity, the dataset should include an ensemble of individual heart/biosignal sets. Ideally, part of the ensemble should be made public as a learning dataset and the other administered by a recognized and reliable institution for purposes of blind testing, i.e. only the MRI data will be provided on request; the calculated BSPM and/or MCG signals will be returned and compared with the publicly undisclosed measured signals. Correlation measures could then act as quality criteria. For the purpose intended here, samples of a few volunteers should be sufficient. At present, heart modelling is not yet as refined as to fully describe biodiversity. However, a means to improve the comparability of models from different sources, should be highly beneficial to improve the status of heart modeling.

The term "reference" is meant in this respect: a unique dataset that can be used and referenced by several modeling groups.

In the following, a dataset is described that could become the basis for such a reference. At present it is designed to serve as an object to stimulate the debate on how a future reference dataset should look, with the hope of stimulating critical and constructive proposals for improvements. An outcome of the debate about this dataset could be to provide proposals for different segmentation algorithms and make their results available as well. Similarly, baseline corrections could be discussed and some of them recommended as an outcome of the debate. But the main objective of this dataset is that the user becomes able to connect dynamic image data - heart and torso geometries - with the electrode and sensor positions where the biosignals were acquired.

## Methods

Five healthy, adult subjects (5 men, mean age 30 ± 5 years) were studied. Informed consent was obtained from all subjects. The study was conducted in accordance with the standards of the Charité and Virchow Hospital Ethics Committee.

The following methods were employed: Magnetic Resonance Imaging (MRI) of heart and torso, Body Surface Potential Maps (BSPM) and MagnetoCardioGraphy (MCG) maps. The latter were recorded simultaneously from the same individuals a few hours after the MRI sessions. Alternatively, one could think of monitoring the BSPM simultaneously during the MRI session. However, it is known, that the ECG signals change in a MRI scanner systematically and considerably due to the magneto-hydrodynamic effect. An MCG cannot be recorded during an MRI session on principle. We believe that our approach - though not perfect - provides a real improvement compared to the present situation.

Although we recorded such datasets for five different volunteers, only a dataset of one individual will be provided here for open access. As will be discussed later, this dataset could serve as a training dataset while the other four may be used for blind testing.

The MR images were taken at the Deutsches Herzzentrum Berlin (DHZB, German Heart Institute Berlin) with a 1.5-T MR Scanner (Philips Intera CV, Best, The Netherlands) equipped with a Nova gradient system (33 mT/m; 160 mT/m/ms). A 5-element cardiac synergy coil was used for signal acquisition. Cardiac cycle synchronization was achieved by using four ECG electrodes placed on the left anterior hemithorax (vector ECG), and scans were triggered on the R wave of the ECG.

**Figure 1:**
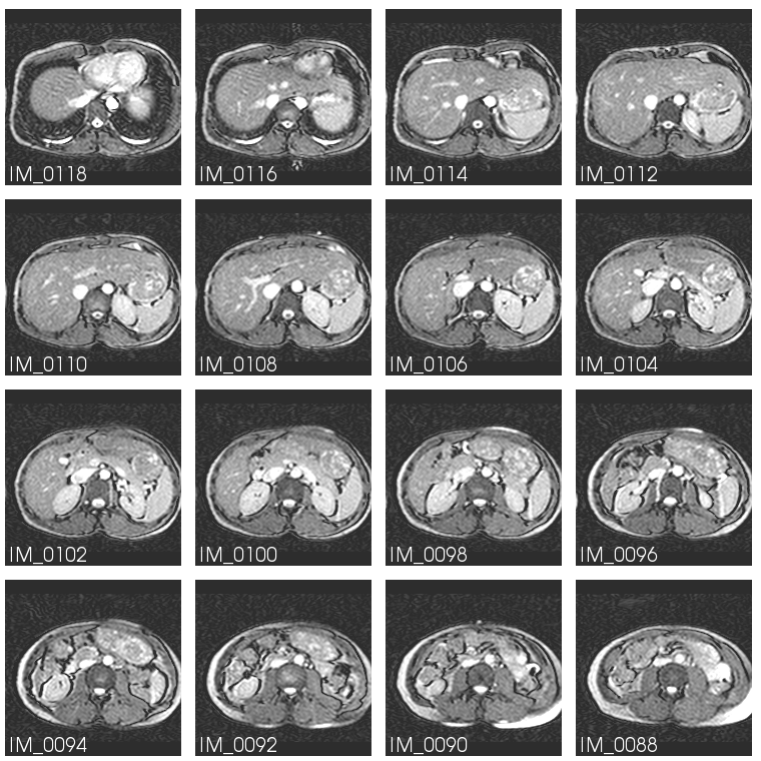
**MR image stack of the torso**. A subset of the survey series (301). IM_0090 and IM_0108 show the marker pills used as spatial reference.

The image series provided for the dataset consist at first of a survey series (series 301). A subset is shown in Figure 1. For this, a breath hold multiple 2D (M2D) single shot balanced Steady State Free Precession (bSSFP) sequence was chosen with the following parameters: TR/TE/flip: 3.1 ms, 1.2 ms, 50 deg; spatial resolution: 3 × 3 × 6 mm^3^; number of slices: 80 (covering 480 mm in feet-head direction); scan time: 21 s. In addition, three further series have been added that contain the dynamic geometry information of the heart. Series 601 (M2D-bSSFP Cine) provides the full heart cycle in several short axis cuts (completely covering the left ventricle); sequence parameters: TR/TE/flip: 3.4 ms, 1.7 ms, 60 deg; spatial resolution: 1.8 × 1.8 × 8 mm^3^; 50 heart phases. The last two series (701 and 801) are free breathing single-phase navigator-gated 3D-bSSFP transversal datasets at diastole and systole, respectively, with the following parameters: T2 preparation (TE = 50 ms) and fat saturation pre-pulses, TR/TE/flip: 4.7 ms, 2.3 ms, 100 deg; measured spatial resolution: 1.5 × 1.5 × 3 mm^3^; reconstructed to 1.5 × 1.5 × 1.5 mm^3^; number of slices: 180 (covering 270 mm in feet/head direction). The acquisition duration per heart beat for the diastolic dataset was 110 ms (scan time: 257 heart beats) and for the systolic dataset 55 ms (scan time: 514 heart beats).

**Figure 2:**
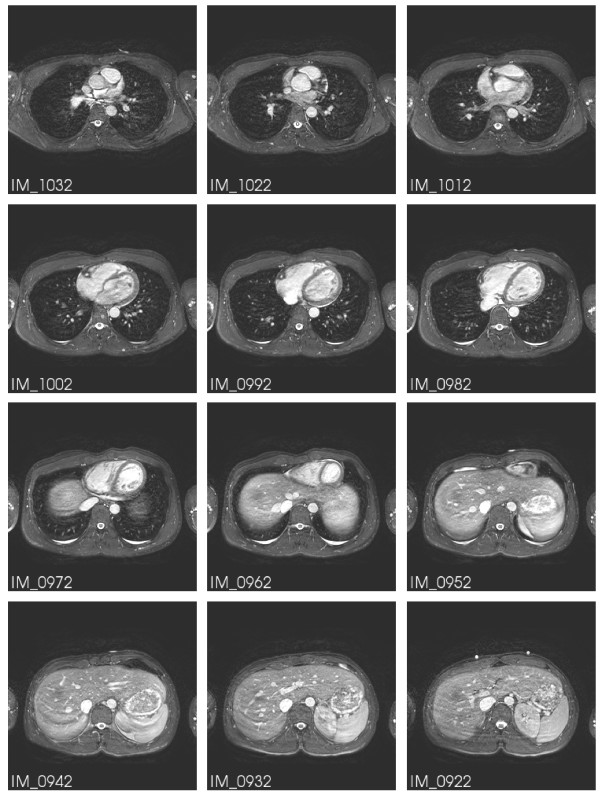
**MR image stack of the heart at diastole**. A subset of series 701. In IM_0922 the same markers are seen as in IM_0108 of Figure 1.

A subset of Series 701 is displayed in Figure 2. IM_0922 shows the same markers as in IM_0108 of Figure 1.

Two types of biosignals have been chosen: i) BSPM and ii) MCG maps. Both provide a broader "field of view" than the conventional ECG and are thus better suited for the intended purpose. Furthermore, in a BSPM the ECG information is practically included.

The reason for adding MCG data lies in the fact that magnetic signals are less influenced by the torso inhomogenities, have no electrode-skin artifacts and are vector quantities (i.e. offer information different from the scalar potential of BSPM).

The biosignals BSP (body surface potential) and MCG (magnetocardiogram) were simultaneously recorded over 100 s with a sampling interval of 1 ms. For the ECG and MCG signals a 20 bit sigma-delta converter and a finite impulse response filter were employed. The latter has a Chebyshev transfer characteristic with linear phase response within the passband from 0 Hz to 350 Hz. Thus the data exhibit typical baseline wander.

**Figure 3:**
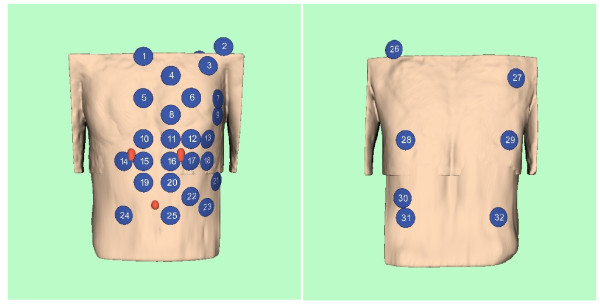
**BSPM lead scheme**. BSPM lead scheme according to Lux et al. [10].

The lead system used to acquire the BSP signals is shown in Figure 3 and follows the scheme introduced by Lux et al. [10].

**Figure 4:**
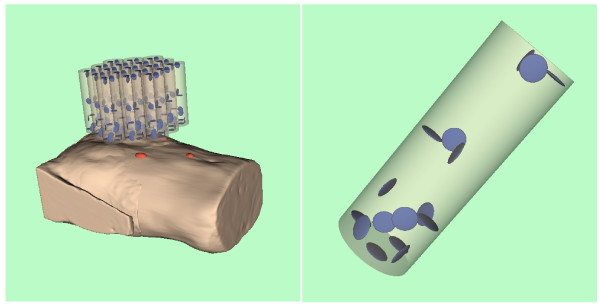
**Detector configuration of the biomagnetic multichannel system**. SQUID configuration consisting of 19 modules; one of the modules schematically shown on right.

The MCG signals were recorded truly synchronously with the BSP signals, with a 304 channel SQUID vector magnetometer system in the Berlin Magnetically Shielded Room II. The SQUID system consists of 19 modules, each containing 16 SQUIDs. Of these, 6 are sensitive to the z component of the magnetic field produced by the heart currents and the other 10 to the perpendicular components. Figure 4 illustrates the configuration.

### Unique coordinate system

**Figure 5:**
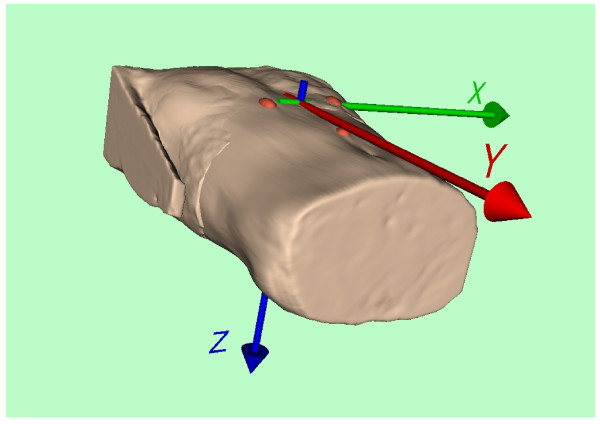
**Coordinate system**. Origin at the middle between the upper two pills.

The different geometries of the three modalities have to be related to a unique coordinate system that is fixed to the individual bodies. Three markers were attached to the volunteer's body. They are clearly visible in the MR images in Figures 1 and 2. The two top markers are vertically aligned and nominally 10 cm apart. The bottom marker is positioned 10 cm below the midpoint between the upper two markers. Figure 5 illustrates their connection to the unique coordinate system chosen:

In the volume reconstruction of the torso generated from the image stacks of series 301 and 701 the three markers are highlighted in red. The origin of the coordinate system was chosen in such a way that it is located in the middle of the two upper pills. The orientations of the x, y, and z axes were chosen according to the conventional Frank ECG lead system [11].

## Results

The training dataset is attached to the supporting online material of this publication. The imaging and biosignal data are raw data, i.e. no segmentation, baseline correction, retrospect filtering or interpolation were performed. Thus it is the choice of the users what methods to consider.

### MRI data

The first image stack (Series 301, Additional file 1), the survey, is included for the purpose of providing information on the reference markers, the torso geometry and the heart position within. Figure 1 displays a subset of this image stack. Note the reference markers in IM_0090 and IM_0108, which are essential for the common coordinate system.

**Figure 6:**
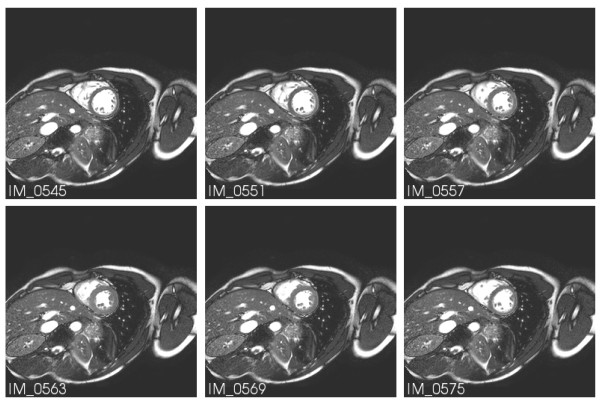
**MR images at different heart cycle phases**. MR images of Series 601 for the time instances marked in Figures 7 and 9 by red cursors.

Series 601 (Additional files 2 and 3) contains for several heart slices the CINE sequence over the whole heart beat period. The images selected in Figure 6 reflect the time instances indicated by red cursors in subsequent figures.

The last two series (701, Additional file 4 and 801, Additional file 5) are the high resolution datasets at diastole and systole, respectively, which should serve as input data for the heart modelling algorithms.

Unfortunately, the raw image data are not the data the modeler wants to know. What is really needed are the volumetric spatial coordinates of the boundaries between different tissue or body compartments, e.g. between blood volume and endocardium, etc. In other words, a thorough segmentation of the image data is an essential processing step to providing the geometrical input data relevant to the model. It is intentional that this segmentation has not been applied to the data here because at present no golden rule for segmentation algorithms exists.

However, a generally well-accepted toolkit for medical image segmentation is ITK. It is available as an open source software package which is widely supported and is thus recommended here [12]. For those not familiar with C^++ ^and installation procedures, a collection of scientific software packages python(x, y) [13] which includes ITK and the visualization toolkit VTK [14] may be easier to use and to install. In addition, ITK and VTK allow reading of the DICOM format and may thus be used for opening the DICOM files of the dataset distribution as well.

For orientation only, a volume reconstruction of the torso from the image stacks of series 301 and 701 was performed using VTK, in order to provide data for the artwork in Figures 3, 4, and 5.

### Biosignal data

The biosignals BSP (body surface potential) and MCG (magnetocardiogram) were simultaneously recorded over 100 s with a sampling interval of 1 ms. A respiration signal was recorded simultaneously and is included in the dataset.

The identifiers and positions of the electrode and sensor centers are given in the accompanying spread sheets in the x, y, and z-coordinates of the unique coordinate system introduced in Figure 5.

It should be noted that the MRI series 601 was acquired in CINE mode. For reference, the instances imaged in Figure 6 are marked by red cursors in the subsequent biosignal plots.

#### BSPM data

**Figure 7:**
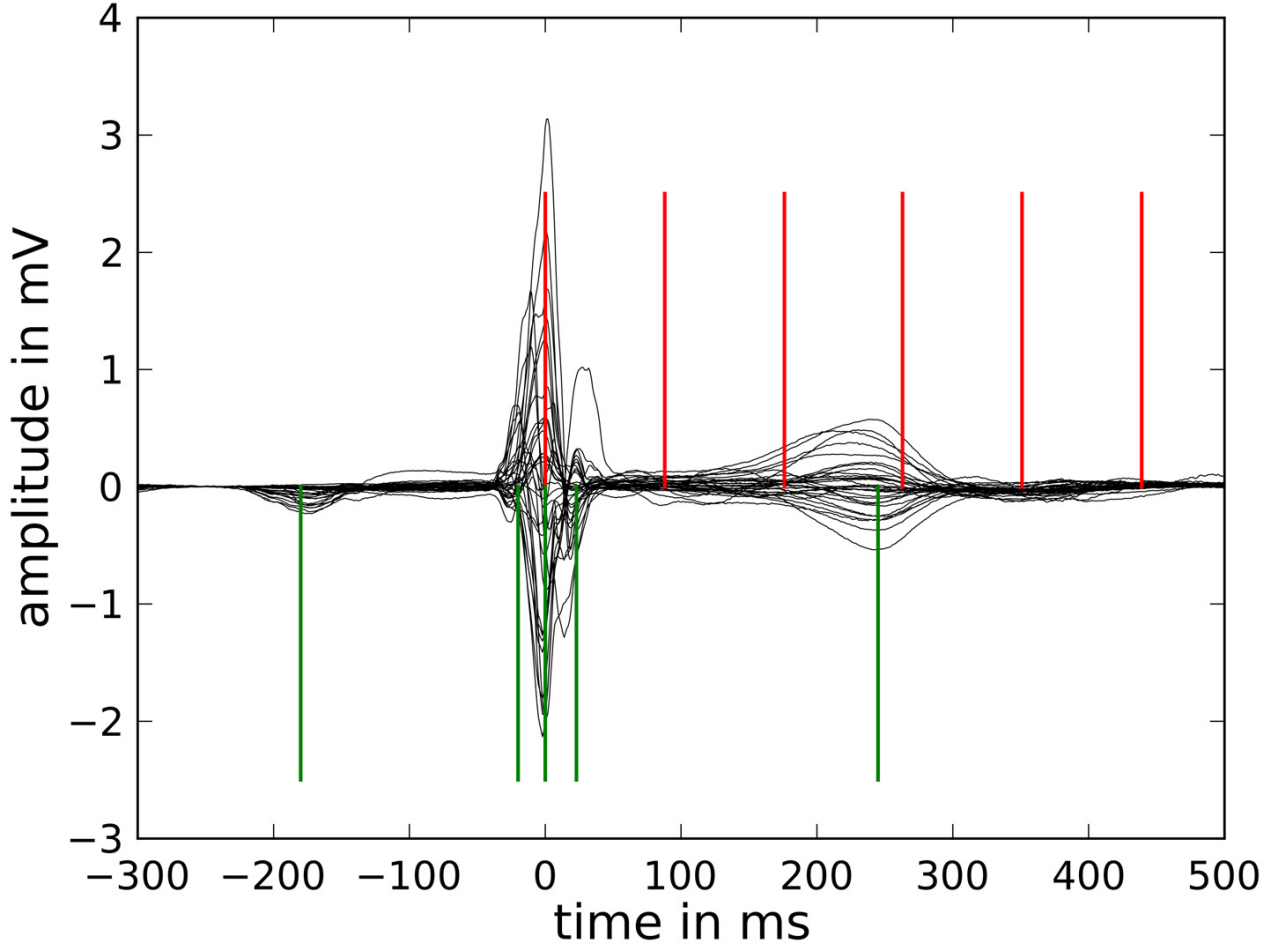
**BSP signals**. Butterfly plot of the averaged signals of all BSP channels. The red cursors mark the time instances of the MR images shown in Figure 6 and the green cursors designate the time instances of the BSP maps in Figure 8.

The BSP signals recorded contained approximately 120 heart beats in the time series (Additional file 6). They were condensed into a single representative beat shown in Figure 7 in a so-called butterfly plot for all BSP channels. The averaging procedure comprised the temporal overlay of all beats centered at the R peak trigger, a moderate baseline correction and a median filter averaging. A detailed description of such an approach is given, e.g., by Koch et al. [15].

Admittedly, the quality of the BSP signals does not represent the state-of-the-art. This is due to the aim to record them truly simultaneously with the MCG signals. This in turn required the use of distinctly non-magnetic ECG electrodes for the BSPM, otherwise the extremely sensitive magnetic recordings would have been deteriorated by artifacts. GRASS F-E5GH electrodes (Astro-Med, Inc.) have proven to contain a very low level of magnetic contamination. However, their small area and only moderate skin contact stability lead to non-optimal ECG signal quality. We aimed to achieve ultimate MCG quality (see next section), as these signals may be better suited for the intended purpose of forward and inverse model verification. Thus the BSP signal quality had to be a compromise.

**Figure 8:**
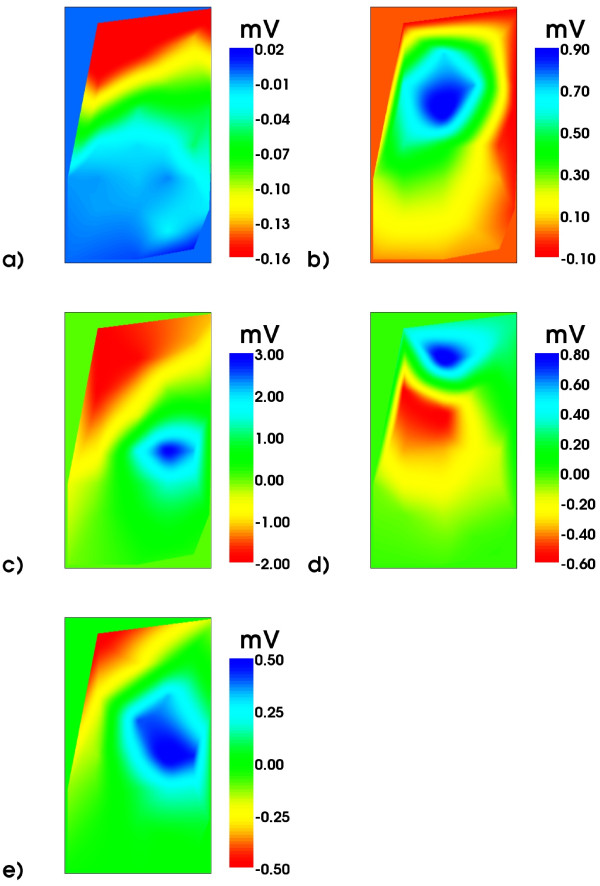
**Body Surface Potential Maps**. BSP maps (only frontal electrodes 1-25): order a), b), ...e) according to the time instances indicated by green cursors in Figure 7. Note the different scales.

From the BSP signals body surface potential maps (BSPM) have been constructed and are shown in Figure 8 for selected time instances marked by the green cursors in Figure 7.

#### MCG data

Magnetocardiography (MCG) is not as well known as its electric counterpart, the ECG. However, for the purpose of model verification it seems to be superior. It is well documented that the magnetic field generated by electrophysiological activity of the heart muscle is far less deteriorated than the respective electric potential at the torso surface. Contrary to the scalar character of an electric potential field, the magnetic equivalent is a vector field. In addition, MCG recordings are contactless and thus do not contain artifacts due to skin-electrode impedance fluctuations.

**Figure 9:**
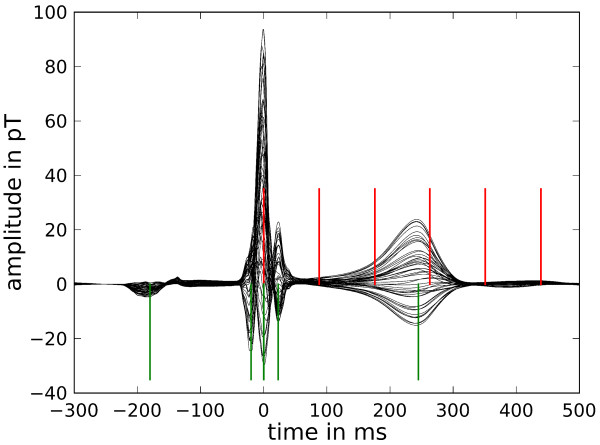
**MCG signals**. Butterfly plot of the lowest level SQUID sensor signals (averaged) with red cursors at time instances of MR images in Figure 6 and green cursors for the time instances of the following maps of the z-component of the MCG signals.

**Figure 10:**
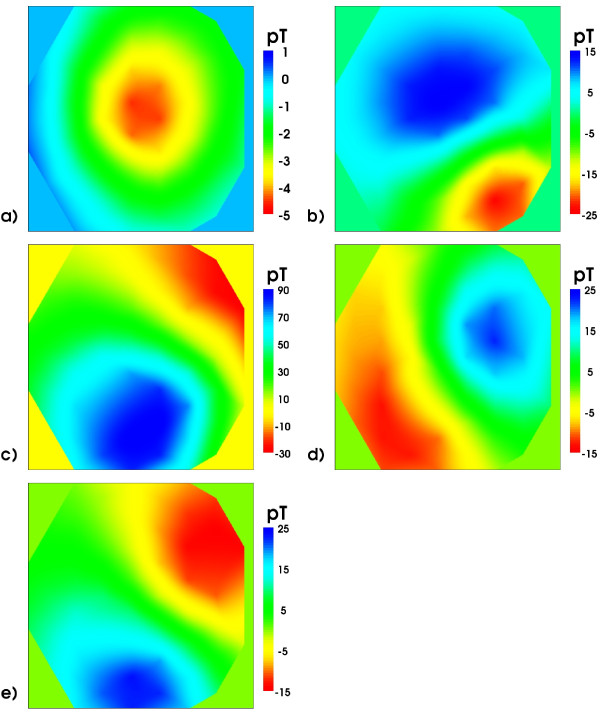
**Magnetic field maps**. Magnetic field maps of the z-component of the magnetic induction signal: order a), b), ...e) according to the time instances marked in Figure 9 by green cursors. Note the different scales. The field patterns at the R peak and T maximum are quite similar for this very regular MCG. Pathological cases would behave rather differently.

The butterfly plot of the MCG is shown in Figure 9 which displays the signals of only 49 channels of the 304 channel system. These stem from the lowest layer of SQUID sensors which measure the z component of the magnetic induction. Figure 9 demonstrates the excellent signal quality of MCG signals. Finally, in Figure 10, the respective MCG maps are shown that correspond to the simultaneously acquired BSPMs shown in Figure 8. The file provided with the attached file folder contains all signals of the system, thus allowing access to the full vector field information (Additional files 7 and 8).

## Discussion

With all the data provided, a verification of numerical electrophysiological heart models could be performed as follows:

• From the respective MRI image sequences, volume geometries of the heart and the torso have to be reconstructed. An adequate software toolkit for this purpose could be VTK [14]. Two small scripts provided as Additional files 9 and 10 respectively show how to access the image data from the Additional files 1, 2, 3, 4, 5.

• These volume reconstructions require segmentation techniques like those provided by ITK [12].

• After these segmentations have been performed successfully, different electric conductivities have to be assigned to the different compartments. The choice of impedance parameters is highly controversial and thus up to the modelers. Inappropriate parameters will lead to considerable discrepancies between forward calculated and measured biosignals. This aspect is one prime motivation for the dataset introduced here.

• These data are then input parameters for the heart model which in turn yields output data on the electric activation of the heart muscle. A very helpful tool for heart modeling, CONTINUITY 6 of the Cardiac Mechanics Research Group of UCSD [16], is made available by the National Biomedical Computation Resource.

• Then a forward calculation of the body surface potentials and/or the resulting magnetic fields should follow.

• Finally, these computed results can be compared to the measured BSPM and/or MCG map data provided by the data files of the web distribution.

MRI series 301 could provide the torso geometries, while series 701 and 801 offer the best resolution data for heart geometries at systole and diastole, respectively.

It has been demonstrated by many heart modelling research groups, that they dispose of all means needed to utilize data such as those provided here to fully implement them in their models. The reviews [17-19] describe these efforts comprehensively. Thus the recipe given above is just a hint how one could proceed. On the other hand, it should not be ignored, that a considerable part of the model quality depends on the acquisition of the heart geometry, i.e. e.g. on the quality of image segmentation. Hence, the approach offered here: raw image data for model input and measured biosignals for comparing model output data, evaluates not only the physiological model algorithm but also these preparatory data handling aspects.

## Conclusions

A comprehensive dataset has been compiled which includes MR image stacks of torso and heart geometries, together with BSPM and MCG data of the same five individuals. One MRI survey series of low resolution provides the geometry of the whole torso. Two high resolution MR image stacks have been acquired for systole and diastole, respectively. Finally, for a few layers of the heart a cine sequence over a full heart cycle has been recorded. All these MRI data offer a wealth of information on the dynamic geometry of an individual heart, which could be utilized as input data for numerical modeling. What makes this dataset particularly valuable are the accompanying biosignal data that were recorded on the same day from the same volunteer. These BSPM and MCG data were acquired simultaneously, i.e. during the MCG recording with a biomagnetic multichannel SQUID system, the BSPM was recorded with ECG electrodes truly in parallel. With this biosignal subset, numerical electrophysiological heart models can be evaluated and verified. For this purpose forward calculations of the body surface potential and/or the magnetic field distribution have to be computed, then a comparison of the calculated with these measured biosignal data may be performed. A procedure commonly used by the heart modelling community [17].

The dataset could also be useful for studies of inverse problem calculations, where epicardial potential or ventricular current density distributions are reconstructed from the BSPM or MCG measurements. For example, whether the reconstructions from BSPM and MCG lead to similar results could be studied.

Although we have recorded such datasets for five different individuals, only one dataset will be provided by open access. This should be useful as a training set to develop the appropriate algorithms. The other four datasets will be reserved for testing purposes upon request.

In that case we would provide the MRI data and the positions of the electrodes and magnetic sensors relative to the reference markers (pills). The client modeler could then return the forward calculated biosignals for the instants of the P, Q, R, S, and T peaks at all electrode/sensor positions to us and we would disclose, how they differ from the respective measured values (for a proper comparison all values will be scaled to the R peak maximum).

## Competing interests

The authors declare that they have no competing interests.

## Authors' contributions

HK contributed substantially to the concept of the dataset, drafted the framework and main part of the manuscript, including the visualizations of the figures, and gave final approval of the version to be published. RDB critically compiled and processed all data. OK performed the simultaneous BSPM and MCG recordings and data handling. BS designed the protocol of the MRI sessions and mediated the technical MR aspects among the partners. CJ, EF, and IP contributed as well to the MRI protocol and performed the MRI acquisition, and gave their approval of the MRI dataset and finally of the manuscript. The authors read and approved the final manuscript.

## Supplementary Material

Additional file 1 **Survey images of the torso**. The image files are in standard DICOM format. For those not familiar with it, a simple commented program script (Additional file 9) is provided. Click here for file

Additional file 2 **First part of torso MRI series in CINE mode**. The image files are in standard DICOM format. For those not familiar with it, a simple commented program script (Additional file 9) is provided. Click here for file

Additional file 3 **Second part of torso MRI series in CINE mode**. The image files are in standard DICOM format. For those not familiar with it, a simple commented program script (Additional file 9) is provided. Click here for file

Additional file 4 **High resolution MRI series of torso with heart at diastole**. The image files are in standard DICOM format. For those not familiar with it, a simple commented program script (Additional file 9) is provided. Click here for file

Additional file 5 **High resolution MRI series of torso with heart at systole**. The image files are in standard DICOM format. For those not familiar with it, a simple commented program script (Additional file 9) is provided. Click here for file

Additional file 6 **BSPM data**. This folder contains: 1. A spreadsheet "BSPMElectrodes.csv" with the electrode positions in mm relative to the origin given by the coordinate system defined by the marker pills (cf. Figure 3). 2. The BSP signals are stored channel-wise in the respective .txt-files of the folder "BSPM_data" with a sampling interval of 1 ms. The signal amplitude values are given as integers and have to be multiplied by a factor of -1.0*10^6 ^in order to obtain potential values in mV. 3. Channel 33 contains the respiration signal. Click here for file

Additional file 7 **First part of MCG data**. This folder contains: 1. A spreadsheet "SquidSensorPosition.csv" with the SQUID sensor positions and orientations within a module in mm and degrees relative to the middle of the bottom plane. 2. A spreadsheet "SquidModulPosition.csv" with the module positions (middle of the bottom plane of the module) in mm relative to the origin given by the coordinate system defined by the marker pills (cf. Figure 3). 3. The MCG signals are stored channel-wise in the respective .txt-files of the MCG_data folders with a sampling interval of 1 ms. The signal amplitude values are given in fT. Click here for file

Additional file 8 **Second part of MCG data**. This folder contains the rest of the MCG data: MCG signals are stored channel-wise in the respective .txt-files of the MCG_data folders with a sampling interval of 1 ms. The signal amplitude values are given in fT. Click here for file

Additional file 9 **For those not familiar with the DICOM format of the files in Additional files **1, 2, 3, 4, 5, **a simple commented program script is provided, demonstrating how to obtain information on various parameters such as patient position and orientation, pixel spacing, etc**. Also shown is how to extract an array of gray scale values of the whole MRI volume covered by the respective session and how to store an image file in txt and png format. In this way, the data are usable for further computations with any software. A precondition for running this program is to install some software toolkits that need to be imported by the programs. The simplest way is to install the full version of python(x, y) http://www.pythonxy.com; everything is then prepared to run the program. Click here for file

Additional file 10 **This program demonstrates an animated volume visualization**. Additional file 9 shows respective snapshots for two different threshold values for the surface extraction module. Noise artifacts are still present, since in this example no sophisticated filtering and segmentation has been adopted. The two 3D-images may be obtained with this program for two different threshold values for "Skin extraction" (top: 80, bottom: 600). A precondition for running this program is to install some software toolkits that need to be imported by the programs. The simplest way is to install the full version of python(x, y) http://www.pythonxy.com; everything is then prepared to run the program. Click here for file
