# Supplementary material for: A reference dataset for verifying numerical electrophysiological heart models
Source: Biomed Eng Online. 2011 Jan 27;10:11. doi: 10.1186/1475-925X-10-11 (PMC3037925; doi:10.1186/1475-925X-10-11)
Supplement: Additional file 4 — High resolution MRI series of torso with heart at diastole. The image files are in standard DICOM format. For those not familiar with it, a simple commented program script (Additional file 9) is provided. [file 1475-925X-10-11-S4.ZIP › MRI_data_set_Series_701/index.htm]

A Reference Data Set for Verifying Numerical Electrophysiological Heart
Models


A Reference Data Set for Verifying Numerical
Electrophysiological Heart Models

---

Additional file folder 1 - MRI data set

Series 701 (Files IM\_0896 - IM\_1075)

| downloadable Files (zip) |
|  |
| Series\_701 |
