# Supplementary material for: A reference dataset for verifying numerical electrophysiological heart models
Source: Biomed Eng Online. 2011 Jan 27;10:11. doi: 10.1186/1475-925X-10-11 (PMC3037925; doi:10.1186/1475-925X-10-11)
Supplement: Additional file 6 — BSPM data. This folder contains: 1. A spreadsheet "BSPMElectrodes.csv" with the electrode positions in mm relative to the origin given by the coordinate system defined by the marker pills (cf. Figure 3). 2. The BSP signals are stored channel-wise in the respective .txt-files of the folder "BSPM_data" with a sampling interval of 1 ms. The signal amplitude values are given as integers and have to be multiplied by a factor of -1.0*106 in order to obtain potential values in mV. 3. Channel 33 contains the respiration signal. [file 1475-925X-10-11-S6.ZIP › BSPM_data_set/index.htm]

A Reference Data Set for Verifying Numerical Electrophysiological Heart
Models


A Reference Data Set for Verifying Numerical
Electrophysiological Heart Models

---

Additional file folder 2 - BSPM data set

BSPM signals and electrode positions

| downloadable Files |
|  |
| electrode positions (csv) |
| BSPM\_data (zip) |
