# Supplementary material for: A reference dataset for verifying numerical electrophysiological heart models
Source: Biomed Eng Online. 2011 Jan 27;10:11. doi: 10.1186/1475-925X-10-11 (PMC3037925; doi:10.1186/1475-925X-10-11)
Supplement: Additional file 7 — First part of MCG data. This folder contains: 1. A spreadsheet "SquidSensorPosition.csv" with the SQUID sensor positions and orientations within a module in mm and degrees relative to the middle of the bottom plane. 2. A spreadsheet "SquidModulPosition.csv" with the module positions (middle of the bottom plane of the module) in mm relative to the origin given by the coordinate system defined by the marker pills (cf. Figure 3). 3. The MCG signals are stored channel-wise in the respective .txt-files of the MCG_data folders with a sampling interval of 1 ms. The signal amplitude values are given in fT. [file 1475-925X-10-11-S7.ZIP › MCG_data_set_Part_I/index.htm]

A Reference Data Set for Verifying Numerical Electrophysiological Heart
Models


A Reference Data Set for Verifying Numerical
Electrophysiological Heart Models

---

Additional file folder 3 - MCG data set - Part I

MCG signals for the SQUID sensors A1, V1 and X1 and the sensor and modul positions

| downloadable Files (zip) |
|  |
| sensor positions (csv) |
| modul positions (csv) |
| MCG signals for channel A1 |
| MCG signals for channel V1 |
| MCG signals for channel X1 |
