# Supplementary material for: A reference dataset for verifying numerical electrophysiological heart models
Source: Biomed Eng Online. 2011 Jan 27;10:11. doi: 10.1186/1475-925X-10-11 (PMC3037925; doi:10.1186/1475-925X-10-11)
Supplement: Additional file 8 — Second part of MCG data. This folder contains the rest of the MCG data: MCG signals are stored channel-wise in the respective .txt-files of the MCG_data folders with a sampling interval of 1 ms. The signal amplitude values are given in fT. [file 1475-925X-10-11-S8.ZIP › MCG_data_set_Part_II/index.htm]

A Reference Data Set for Verifying Numerical Electrophysiological Heart
Models


A Reference Data Set for Verifying Numerical
Electrophysiological Heart Models

---

Additional file folder 3 - MCG data set - Part II

MCG signals for the SQUID sensors Z1, Z2 and Z3

| downloadable Files (zip) |
|  |
| MCG signals for channel Z1 |
| MCG signals for channel Z2 |
| MCG signals for channel Z3 |
