# Supplementary material for: A reference dataset for verifying numerical electrophysiological heart models
Source: Biomed Eng Online. 2011 Jan 27;10:11. doi: 10.1186/1475-925X-10-11 (PMC3037925; doi:10.1186/1475-925X-10-11)
Supplement: Additional file 9 — For those not familiar with the DICOM format of the files in Additional files 1, 2, 3, 4, 5, a simple commented program script is provided, demonstrating how to obtain information on various parameters such as patient position and orientation, pixel spacing, etc. Also shown is how to extract an array of gray scale values of the whole MRI volume covered by the respective session and how to store an image file in txt and png format. In this way, the data are usable for further computations with any software. A precondition for running this program is to install some software toolkits that need to be imported by the programs. The simplest way is to install the full version of python(x, y) http://www.pythonxy.com; everything is then prepared to run the program. [file 1475-925X-10-11-S9.PDF]

Demo program 1

```
"""

Created on Mon Mar 08 13:50:52 2010


@author: Hans Koch

"""


# This example reads a volume dataset using VTK, exports the gray scale values

# to a numpy array, saves a sub-array into a csv-file and extracts a png-image


# The following modules have to be imported:


import numpy as np

import vtk

from PIL import Image


# Reading the DICOM files:


reader = vtk.vtkDICOMImageReader()

reader.SetDirectoryName('E:/series_701/')   # use your respective path!

reader.Update()


# …following are some examples of  how to gain information:
```

```
print 'data extent = ', reader.GetDataExtent()

print 'pixel spacing = ', reader.GetPixelSpacing()

print 'image position patient = ', reader.GetImagePositionPatient()

print 'image orientation patient = ', reader.GetImageOrientationPatient()


# How to extract the DICOM image stack into a NumPy array:


dim = np.array(reader.GetDataExtent(), dtype=int) +1

a = np.zeros((dim[5], dim[3], dim[1]), np.float32)


exprt = vtk.vtkImageExport()

exprt.SetInputConnection(reader.GetOutputPort())

exprt.ImageLowerLeftOn()

exprt.Export(a)


# Now "a" is an array that contains all images of series 701

# Please note that the gray scale values for each pixel are floating point values

# with a range from 0.0 till well over 2000.0!


# Save a selected part (in this example: layer 50, rows 110 to 120 and

# columns 90 to 100) of this array to a csv-file

# which is easily readable by most programs:


np.savetxt('E:/mrt_701.csv', a[50,110:120,90:100], fmt = '%6.2f', delimiter = ',')
```

```
# How to extract a png image for, let's say, the image in layer 50:

# The gray scale has to be adjusted and converted to integers


ar = a[50,:,:]/6.0

b = np.where((ar < 255),ar,255)

img = np.array(b, np.int8)


pngImage = Image.fromarray(img)

pngImage.save('E:/mrt_701.png')


# Here is the saved png image!

# Of course one could have obtained it more directly, but the intention above

# was to demonstrate the export to an array and to extract an image from that.
```
