# Supplementary material for: A reference dataset for verifying numerical electrophysiological heart models
Source: Biomed Eng Online. 2011 Jan 27;10:11. doi: 10.1186/1475-925X-10-11 (PMC3037925; doi:10.1186/1475-925X-10-11)
Supplement: Additional file 10 — This program demonstrates an animated volume visualization. Additional file 9 shows respective snapshots for two different threshold values for the surface extraction module. Noise artifacts are still present, since in this example no sophisticated filtering and segmentation has been adopted. The two 3D-images may be obtained with this program for two different threshold values for "Skin extraction" (top: 80, bottom: 600). A precondition for running this program is to install some software toolkits that need to be imported by the programs. The simplest way is to install the full version of python(x, y) http://www.pythonxy.com; everything is then prepared to run the program. [file 1475-925X-10-11-S10.PDF]

Demo program 2

```
"""

Created on Mon Mar 08 13:50:52 2010


@author: Hans Koch

"""


# This example reads a volume dataset using vtk, renders and animates it


import vtk


# Reading the DICOM files:


reader = vtk.vtkDICOMImageReader()

reader.SetDirectoryName('E:/series_701/')   # use your respective path!

reader.Update()


# volume visualization (module names are self-explanatory):


skinExtractor = vtk.vtkContourFilter()

skinExtractor.SetInput(reader.GetOutput())

skinExtractor.SetValue(0, 80)

# Selection of this threshold value determines what surfaces become visualized.

# Play with it!
```

```
skinMapper = vtk.vtkPolyDataMapper()

skinMapper.SetInputConnection(skinExtractor.GetOutputPort())

skinMapper.ScalarVisibilityOff()

skinActor = vtk.vtkActor()

skinActor.SetMapper(skinMapper)

ren = vtk.vtkRenderer()

ren.AddActor(skinActor)

ren.SetBackground(1, 1, 1)

renWin = vtk.vtkRenderWindow()

renWin.AddRenderer(ren)

renWin.SetSize(500, 500)

iren = vtk.vtkRenderWindowInteractor()

iren.SetRenderWindow(renWin)

iren.Initialize()

renWin.Render()

iren.Start()


# converts the window content to an image:


w2i = vtk.vtkWindowToImageFilter()

w2i.SetInput(renWin)


writer = vtk.vtkJPEGWriter()
```

```
writer.SetInput(w2i.GetOutput())

writer.SetFileName('E:/volume_80.jpg')

writer.Write()
```

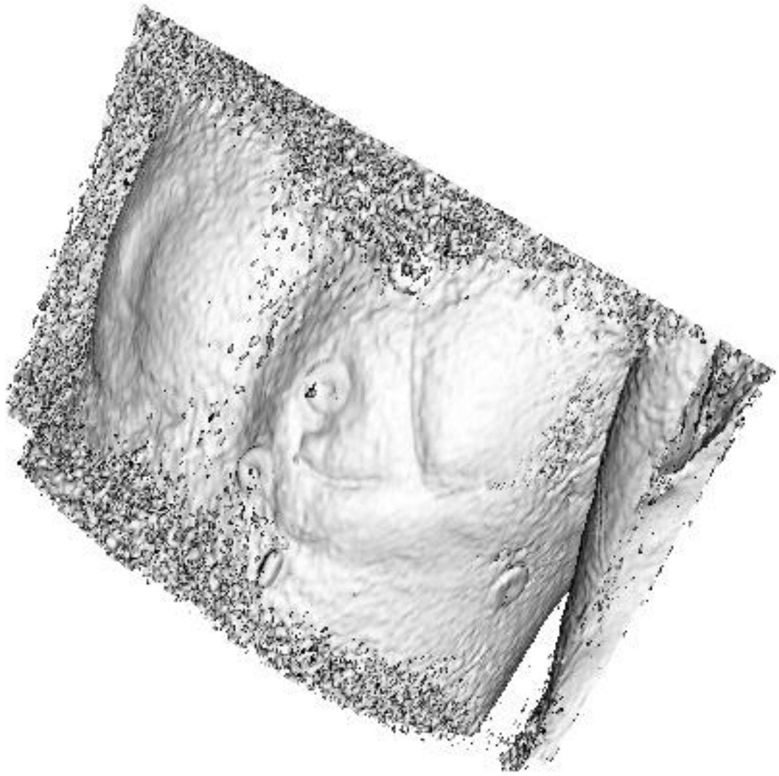

Output for skinExtractor.SetValue(0, 80)

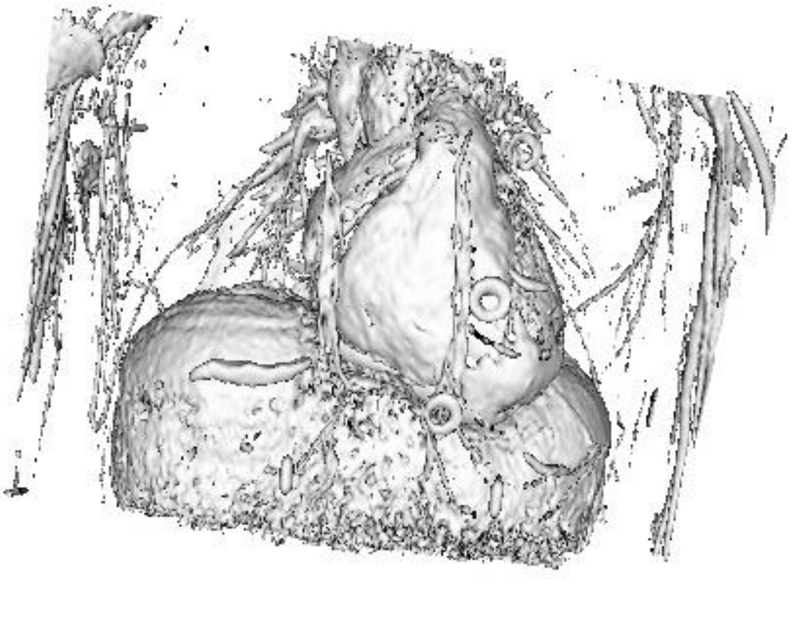

Output for skinExtractor.SetValue(0, 600)
